# Role of Prostaglandins in Neuroinflammatory and Neurodegenerative Diseases

**DOI:** 10.1155/2012/946813

**Published:** 2012-06-18

**Authors:** Isabel Vieira de Assis Lima, Leandro Francisco Silva Bastos, Marcelo Limborço-Filho, Bernd L. Fiebich, Antonio Carlos Pinheiro de Oliveira

**Affiliations:** ^1^Department of Pharmacology, Federal University of Minas Gerais, Avenida Antonio Carlos, 6627, 31270-901 Belo Horizonte, MG, Brazil; ^2^Department of Physiology and Biophysics, Federal University of Minas Gerais, Avenida Antonio Carlos, 6627, 31270-901 Belo Horizonte, Brazil; ^3^Department of Psychology and Neuroscience, Muenzinger Building, Colorado University of Colorado Boulder, Avenida, Boulder, CO 80309-0354, USA; ^4^Department of Psychiatry and Psychotherapy, University of Freiburg Medical School, Hauptstraße 5, 79104 Freiburg, Germany; ^5^VivaCell Biotechnology GmbH, Ferdinand-Porsche-Straße 5, 79211 Denzlingen, Germany

## Abstract

Increasing data demonstrates that inflammation participates in the pathophysiology of neurodegenerative diseases. Among the different inflammatory mediators involved, prostaglandins play an important role. The effects induced by prostaglandins might be mediated by activation of their known receptors or by nonclassical mechanisms. In the present paper, we discuss the evidences that link prostaglandins, as well as the enzymes that produce them, to some neurological diseases.

## 1. Neuroinflammation and Neurodegeneration

Neuroinflammation plays a key role in the progression or resolution of pathological conditions. Inflammatory responses in the brain parenchyma have been associated with the etiopathogenesis of different neurological disorders, including central nervous system (CNS) infection, brain ischemia, multiple sclerosis, Alzheimer's disease, and Parkinson's disease [1–7]. Then, it is presently clear that neuroinflammation is a key feature shared by many neurodegenerative disorders [8, 9].

Different CNS cells, such as microglia, astrocytes, oligodendrocytes, and neurons produce a plethora of inflammatory mediators, which act either in a paracrine or an autocrine fashion, leading to an intricate cross-talk between these different cell types. Among these mediators, many studies have demonstrated that CNS cells produce prostanoids and that these mediators might contribute to the normal CNS function or to enhance the neuroinflammatory and neurodegenerative processes [10]. Herein, we review the current knowledge on the role of prostaglandins, as well as the enzymes that synthesize them, in neuroinflammatory and neurodegenerative diseases.

## 2. Roles of Prostaglandins in Neuroinflammation: *In Vitro* and *In Vivo* Evidences

Due to the variety of prostaglandins presently known, it is reasonable to speculate that these lipid mediators might play different roles in the CNS. Below, we describe some *in vivo* and *in vitro* data with regard to the potential role of specific prostanoids in neuroinflammation. 

### 2.1. PGE_2_

To date, three prostaglandin (PG) E synthases (PGESs) have been characterized: the microsomal PGESs (mPGES-1 and mPGES-2) and the cytosolic PGES (cPGES) [11–14]. mPGES-1 is an inducible enzyme and is expressed also in activated microglia [15, 16]. There are at least four characterized PGE_2_ receptors, namely, EP1, EP2, EP3, and EP4. This prostaglandin modulates the expression of inflammatory mediators by microglial cells. For example, PGE_2_ and EP agonists inhibited the expression of inducible nitric oxide synthase (iNOS) and nitric oxide (NO) generation [17] and enhanced the expression of cyclooxygenase (COX)-2 induced by lypopolysaccharide (LPS) in cultured microglia [18]. Moreover, an EP2 agonist inhibited interleukin (IL)-1*β* release by cultured primary rat microglia stimulated with LPS, although no reduction of this cytokine was observed with EP1, EP3, and EP4 agonists [19].

Intraperitoneal injection of LPS increased the expression of EP4 receptors in microglial cells and in the hippocampus of mice [20]. Interestingly, activation of EP4 receptors reduced the expression of different cytokines, COX-2 and iNOS in BV-2 and primary mouse microglial cells [20].

### 2.2. PGD_2_

PGD_2_ has also been shown to be important in neuroinflammatory conditions. A 6-day infusion of LPS in the fourth cerebral ventricle of rats enhanced the PGD_2_ production in the brain [21]. It has been shown that PGD_2_ produced by microglia acts on DP1 receptors of astrocytes, leading to astrogliosis. Moreover, oligodendroglial apoptosis was reduced by hematopoietic prostaglandin D synthase (HPGDS) inhibitor and in HPGDS-null mice, suggesting an important effect of PGD_2_ in demyelination in twitcher mice, a model of Krabbe disease [22]. Expression of DP1 and HPGDS is also increased in the brains of patients with Alzheimer's disease [23].

PGD_2_ also induced apoptosis of mouse oligodendrocyte precursor (mOP) cells, what could interfere in the demyelination process that occurs in multiple sclerosis [24]. It was shown that mice deficient in lipocalin-PGDS reveal an increased number of apoptotic neurons and olygodendrocytes, suggesting a protective role of lipocalin-type PGDS in the genetic demyelinating mouse twitcher [25].

### 2.3. 15-Deoxy-Δ^12,14^-Prostaglandin J_2_ (15d-PGJ_2_)

15d-PGJ_2_ is a metabolite of PGD_2_ and is formed from PGD_2_ by the elimination of two molecules of water. At least some effects mediated by 15d-PGJ_2_ are mediated by activation of the peroxisome proliferator-activated receptors (PPARs)*γ*. This prostaglandin has been shown to inhibit NO and tumor necrosis factor (TNF)-*α* production as well as expression of major histocompatibility complex (MHC) class II in activated microglia, suggesting that this prostaglandin might be important to modulate microglia functions [26]. Similar effects, such as downregulation of iNOS and cytokines, have also been observed in astrocytes [27].

### 2.4. PGI_2_

Few studies were carried out to investigate the role of PGI_2_ in the CNS. In general, these studies suggest a neuroprotective role for PGI_2_ against different stimuli. For example, enhancement of PGI_2_ synthesis in neuron-glia cultures by adenoviral gene transfer of PGI synthase (PGIS) reduces the expression of different inflammatory mediators induced by LPS, such as TNF-*α* [28], and PGI_2_ receptor ligands prevented the death of hippocampal neurons induced by high oxygen, xanthine + xanthine oxidase, or serum deprivation [29]. Interestingly, 15-deoxy-(16-m-tolyl)-17,18,19,20-tetranorisocarbacyclin methyl ester, a selective central type PGI_2_ receptor ligand, reduced brain damage induced by middle cerebral artery occlusion [30].

### 2.5. PGF_2*α*_

In rat primary neuronal culture, hypoxia increased PGF_2*α*_ content. Importantly, previous addition of this prostaglandin to the culture medium exacerbated hypoxic injury [31]. PGF_2*α*_ reduced TNF-*α* in primary spinal cord cultures stimulated with LPS [32]. In a model of unilateral middle cerebral artery occlusion, knockout (KO) mice to FP, the receptor for PGF_2*α*_, have less neurological deficit and smaller infarct volumes [33]. The KO animals were also less sensitive to excitotoxicity induced by unilateral intrastriatal N-methyl-D-aspartate injection. In agreement with that, in the same model, the FP agonist latanoprost increased neurological deficit and infarct size in wildtype (WT) mice [33].

## 3. Roles of Prostaglandins in Neurodegenerative Diseases

As previously mentioned, there are strong evidences that inflammation contributes to etiopathogenesis of neuroinflammatory and neurodegenerative diseases. Below, we discuss the involvement of prostaglandins in these neuropathological conditions.

### 3.1. Multiple Sclerosis (MS)

A neuroinflammatory component is very evident in the etiopathogenesis of MS. MS is an autoimmune demyelinating disorder characterized by distinct episodes of neurologic deficits attributable to white matter lesions. It is the most common of the demyelinating disorders, which affects predominantly northern Europeans. The disease becomes clinically apparent at any age, although onset in childhood or after 50 years of age is relatively rare. Women are affected twice as often as men. In most individuals with MS, the illness shows relapsing and remitting episodes of neurologic deficits. The frequency of relapses tends to decrease during the course of the disease, but there is a steady neurologic deterioration in a subset of patients [34].

Modeling clinical aspects of any human disease in rodents and cells is a big challenge in all fields of research. However, it is especially more challenging to model MS, because this is an exclusively human disease, its etiopathogenesis is unknown, and this disease is multifaceted, which occur in a relapsing-remitting manner. As the toxin-induced models of demyelination such as those induced by cuprizone, ethidium bromide and lysolecithin are important to understand demyelination and remyelination but do not resemble the human disease as efficiently as the autoimmune model (experimental autoimmune encephalomyelitis EAE), this paper will be focused on the roles played by prostaglandins in this model because of its presumed higher predictive validity [35].

#### 3.1.1. Phospholipase A_2_ (PLA_2_) and COX

There is a large body of evidence demonstrating the role played by prostanoids in the onset and progression of EAE in a wide variety of animal models as well as in *in vitro* studies. Within the last decade, some studies have demonstrated that cytosolic PLA_2_ (cPLA_2_) plays a key role in the etiopathogenesis of EAE [36–39]. There are evidences supporting distinct roles played by different isoforms of PLA_2_ in the onset or progression of EAE [40]. cPLA_2_ plays a role in the onset of EAE, calcium-independent PLA_2_ in the onset and progression, and secretory type II PLA_2_ in the later remission phase. Immunohistochemical labeling of cPLA_2_ was shown in either immune or endothelial cells in the spinal cord lesions of mice with EAE induced by myelin oligodendrocyte glycoprotein (MOG). Both preemptive and therapeutic treatments with a selective cPLA_2_ inhibitor resulted in marked reduction in the onset and progression of EAE. Accordingly, the reduced clinical score parallels with reduced spinal protein concentration of COX-2 and both gene expression and protein concentrations of dozens of inflammatory mediators, including several cytokines and chemokines which are implicated with the etiopathogenesis of EAE [36]. Moreover, selective inhibition of cPLA_2*α*_ prevents EAE and suppresses Th1 and Th17 responses [38]. cPLA_2*α*_ inhibitors diminish the ability of antigen-presenting cells to induce antigen-specific effector T-cell proliferation and inflammatory cytokine production, inhibit microglial activation, and increase oligodendrocyte survival [39]. The latter study also showed that if cPLA_2*α*_ inhibitors are administered at the peak of disease or during remission—relapsing-remitting model—, the subsequent relapse is abolished. Consistently with these pharmacological studies, a genetic study showed that cPLA_2*α*_-deficient mice are resistant to EAE [37].

COX-1 and -2 are upregulated in the CNS of animals in different EAE models [36, 38, 41]. Accordingly, different selective and nonselective inhibitors of COX isoforms induce beneficial effects in different animal models of EAE. EAE onset is delayed if diet is supplemented with acetylsalicylic acid shortly after its induction in Lewis rats [42]. Indomethacin, another non-selective COX inhibitor, attenuates the progression of EAE [43].

#### 3.1.2. PGE_2_

PGE_2_ seems to be the eicosanoid which is more strongly implicated with EAE onset and progression. Bolton and colleagues investigated the CNS concentrations of PGE_2_, 6-oxo-PGF_1*α*_, and PGF_2*α*_ in acute EAE-affected guinea pigs [44]. They showed that a PGE_2_ concentration increase in spinal cord and cerebellum precedes EAE onset, whereas the other two prostanoids were found to peak after the observation of the first clinical signs of EAE. The behavioral syndrome associated with EAE is also preceded by increased CNS concentration of PGE_2_ in mice [45]. A wide screening that examined the correlation between many arachidonic acid (AA) pathway products and EAE onset and progression showed that PGE_2_ (concomitantly with its receptors EP1, EP2, and EP4) is synthesized more markedly than other eicosanoids [46], suggesting an important role in exacerbating EAE. However, dual roles played by PGE_2_ have been recently shown in mouse EAE. PGE_2_ exacerbates Th1 and Th2 responses via EP2 and EP4 receptors during mouse EAE onset and protects the brain from immune cell infiltration via EP4 receptor [47].

mPGES-1 upregulation occurs in microglia/macrophages in the spinal cord lesions of mice with EAE induced by MOG as well as in brain tissues from MS patients. mPGES-1-deficient mice exhibit a better clinical score and suppressed Th1 and Th17 responses when compared with those of nongenetically modified control mice after EAE induction [46]. Regarding the untoward gastric and cardiovascular effects induced by COX inhibitors [48], there is an eagerness to discover compounds that target mPGES-1 for treating inflammatory diseases [49–51] because this enzyme is downstream to COX-2 in AA pathway.

#### 3.1.3. 15d-PGJ_2_

Systemic treatment with 15d-PGJ_2_ inhibits EAE progression in mice, and this is associated with reduced demyelination, neuroinflammation, IL-12 production by macrophage/microglial cells, T-cell proliferation, and IL-12-induced T-cell responses [52]. Moreover, pretreatment with this agonist of PPAR*γ* delays the onset of EAE and reduces the spinal cord infiltration of CD4^+^ T cells and macrophages [53]. 15d-PGJ_2_ suppresses the production of cytokines and/or chemokines in cultured T cells, microglia, and astrocytes [53–55]. Providing further support to the role played by 15d-PGJ_2_ in EAE etiopathogenesis, it was shown that PPAR*γ* antagonists reverse the inhibition of EAE clinical signs and Th1 response by this cyclopentanone prostaglandin [56].

#### 3.1.4. Other Prostaglandins

As there is a correlation between increased spinal PGDS concentration and the initiation of relapsing phase of EAE, it has been suggested a role played by this isomerase in this phenomenon [57]. Indeed, PGD_2_ is released from mast cells in allergic reactions, and it is suggested to modulate allergic inflammation [58, 59]. On the other hand, a more recent study showed that PGD_2_, PGI_2_ and 5-lipoxygenase pathways are suppressed in the acute phase of EAE and returns to constitutive levels in the chronic phase [46]. However, in a relapsing-remitting model, PGD_2_ remained unaffected throughout all phases [41].

### 3.2. Alzheimer's Disease (AD)

The first evidences supporting a role played by inflammation on AD onset rose up in the late 1980s, when many signs of inflammation in *postmortem* brains from AD patients were observed, such as activated lymphocytes and microglial cells in plaque and tangle lesions, presence of complement proteins, cell lysis, and opsonisation of debris [60–64].

#### 3.2.1. PLA_2_ and COX

It was hypothesized that the long-term use of nonsteroidal anti-inflammatory drugs (NSAIDs) could reduce the risk for AD or delay disease onset. Indeed, McGeer et al. [65] observed a clear negative correlation between the prevalence of AD in general population versus that in rheumatoid arthritis patients taking NSAIDs, mainly salicylates. Reinforcing this evidence, a clinical trial conducted shortly afterwards, showed that treatment with indomethacin, a nonselective COX inhibitor, improves cognitive deficits in AD patients [66]. Since then, epidemiological studies have been showing either beneficial or detrimental effects induced by COX inhibitors on AD risk and delay of onset, though beneficial effects are mostly observed [67]. Despite controversy, these studies clearly show that prostanoids play an important role in AD etiopathogenesis.

cPLA_2_, which cleaves AA from cellular membrane phospholipids, is elevated in AD brain [68]. The cyclooxygenation and subsequent isomerization of AA produces prostaglandins, which regulate immune responses and neurotransmission [69, 70]. Accordingly, increased expression of COX-1 and -2 is observed in AD-affected brains [71, 72]. One of the most versatile products of this cascade is PGE_2_, which is produced by glial cells and neurons.

#### 3.2.2. PGE_2_

An increased expression of mPGES-1 and mPGES-2 is observed in the brain of AD brains [73, 74]. Moreover, patients with probable AD have higher cerebrospinal fluid (CSF) concentrations of PGE_2_ than age-matched control subjects [75]. It has been shown that PGE_2_ increases amyloid precursor protein (APP) gene expression and production *in vitro* [76–78]. This effect is inhibited by immunosuppressants in astrocytes [77] and is associated with EP2 receptor activation in microglial cells [78]. On the other hand, there is evidence supporting an anti-inflammatory role played by PGE_2_ mediated by EP4 receptor in LPS-stimulated cultured microglial cells [20]. However, PGE_2_ increases APP production via both EP2 and EP4 receptors (but not via EP1 and EP3 ones) both *in vitro* and *in vivo* [76, 79]. Hoshino et al. [76] showed that PGE_2_-dependent internalization of EP4 receptor increases *γ*-secretase activity, which in turn leads to higher proteolysis of APP.

In transgenic mice overexpressing APP, selective inhibition of COX-2 blocks amyloid *β* (A*β*)-induced suppression of hippocampal long-term potentiation (LTP) and memory function independently of reductions in A*β*42 and inflammatory cytokines, but markedly dependent on PGE_2_ concentrations, showing an additional mechanism by which NSAIDs may protect against AD progression and an important synaptic role of PGE_2_ in this setting [80]. EP2 receptors are important mediators of PGE_2_ actions on electrophysiological properties of hippocampal neurons, as EP^−/−^ mice exhibit cognitive deficits in social memory tests associated with a deficit in long-term depression in hippocampus [81]. Pharmacological studies corroborate these previously mentioned findings. Either exogenous or endogenous PGE_2_, but not exogenously applied PGD_2_ or PGF_2*α*_, regulates hippocampal neuronal plasticity [69, 70].

#### 3.2.3. PGD_2_ and 15d-PGJ_2_

One of the first studies which assessed prostaglandins concentrations in *postmortem* cerebral cortices of probable AD patients showed that only PGD_2_ was increased in comparison with age-matched control subjects [82]. Indeed, PGDS expression was found to be localized in microglial cells surrounding senile plaques, and DP1 receptor expression was observed in microglial cells and astrocytes within senile plaques in human AD brains. In Tg2576 transgenic mice—a model of AD disease—, the DP1 receptor expression increases in parallel with A*β* deposition [23].

As 15d-PGJ_2_ induces neuronal apoptosis [83], it was initially suggested that this prostanoid is associated with neurodegeneration. However, it was shown afterwards that 15d-PGJ_2_ reduces microglial production of NO, IL-6, and TNF-*α* induced by A*β*40, which suggests anti-inflammatory indirect neuroprotective effect [84]. Accordingly, not only 15d-PGJ_2_, but also troglitazone and ciglitazone, other compounds known to activate PPAR*γ* and attenuate the A*β*-induced impairment of hippocampal LTP *in vitro*, supporting a possible beneficial effect on AD progression.

### 3.3. Parkinson's Disease (PD)

PD is the second most common neurodegenerative disease, characterized by abnormal motor symptoms such as stiffness, postural instability, slowness of movement, resting tremor, and bradykinesia. The neuropathological features of PD are progressive death of dopaminergic neurons in the substantia nigra (SN) pars compacta that project to the striatum. The exact cause of this cell death is not clear, but recent studies have shown that the process may involve inflammatory reactions, in addition to oxidative stress, mitochondrial dysfunction, neural excitotoxicity, and insufficient neurotrophic factors [85–87].

It is known that, in the SN of PD brains, microglia is activated [5], and its activation has been strongly associated with CNS pathology of PD, by production of proinflammatory and cytotoxic factors, such as cytokines, chemokines, NO, reactive oxygen species (ROS), and AA metabolites [88, 89].

#### 3.3.1. PLA_2_ and COX

It has been shown that mice carrying a mutation of the cPLA_2_ gene, leading to an absence of cPLA_2_ activity, are resistant to 1-methyl-4-phenyl-1,2,3,6-tetrahydropyridine (MPTP), a precursor to 1-methyl-4-phenylpyridinium (MPP^+^) -induced neurotoxicity, a well-known model of PD [90]. Dopamine receptor activation also increased cPLA_2_ activity in a rat model of PD [91].

Many data demonstrated also an alteration of COX-2 expression in PD. In fact, different studies have shown an upregulation of COX-2 in animal models of PD [92–94]. COX-2 increased expression has been also demonstrated in the SN of *postmortem* PD specimens in comparison to normal controls [95, 96]. Moreover, it has been shown that COX inhibition [93, 94, 97, 98] and COX transgenic ablation [99–101] in *in vivo* models of PD increased survival of dopaminergic neurons. However, this effect was not observed in all studies. Rofecoxib, a COX-2 inhibitor, did not change MPTP-induced neurodegeneration and, paradoxically, caused a significantly augmented basal prostaglandin production [92].

Regular use of NSAIDs is associated with a lower risk of PD compared with nonregular users of these drugs [85, 102]. However, this is still controversial, since recent studies could not demonstrate a protective effect of NSAIDs in PD [103–105]. Considering that these drugs might have other mechanisms of action unrelated to COX inhibition, it is important to evaluate the effect of specific compounds in the prevention or treatment of PD.

#### 3.3.2. PGE_2_

It has been observed that PGE_2_ is significantly elevated in the CSF and SN of PD patients in comparison to control subjects. Moreover, incubation of slices of SN with AA induced an increased production of PGE_2_ synthesis, suggesting an enhancement of the enzymes responsible to its production [106].

Release of aggregated *α*-synuclein, a major component of Lewy bodies in PD, after neuronal damage, may activate microglia. This activation could, in turn, lead to production of proinflammatory mediators, such as PGE_2_ [107], contributing to the progression of nigral neurodegeneration. A pretreatment of primary mesencephalic neuron-glia mouse cultures with *α*-synuclein enhances the production of PGE_2_. Apparently, phagocytosis of *α*-synuclein activates NADPH oxidase, which produces ROS, and has a crucial role in microglial activation and associated neurotoxicity [107].

In primary mesencephalic mixed neuron-microglia cultures, MPP^+^, a neurotoxin that causes dopaminergic neuronal death, induced PGE_2_ production. However, this effect was not observed in enriched microglia and enriched neuron cultures, indicating that is necessary an interaction between microglia and neurons for MPP^+^-induced increase of the PGE_2_ production, probably due to COX-2 activity. Moreover, PGE_2_ was not enhanced neither in enriched astroglia nor in neuron-astroglia cultures [94]. Conversely, PGE_2_ was significantly reduced in the hippocampus, striatum, and cortex of animals injected with 6-hydroxydopamine (6-OHDA) [108].

It has been shown that EP receptors are expressed differently in the SN. To date, in the rat, EP1 is restricted to dopaminergic neurons, while EP3 is expressed exclusively by nondopaminergic cells. On the other hand, EP2 is localized to both dopaminergic and nondopaminergic cells [109]. In rats, EP1, but not EP2 and EP3 receptor antagonists, reduced the dopaminergic neuronal death induced by 6-OHDA, suggesting an important effect of EP1 receptor in the neurotoxicity induced by PGE_2_ [109]. Also, culture of dopaminergic neurons displayed EP2 receptors after 6-OHDA neurotoxicity, and butaprost, a selective EP2 agonist, significantly increased survival of tyrosine hydroxylase positive cells, suggesting a possible neuroprotective role of EP2 of activation [110].

Interestingly, in comparison to microglia obtained of WT animals, microglia of EP2 KO mice reveal an enhanced capacity to clear aggregated *α*-synuclein in human mesocortex tissue of patients with Lewy body disease. Moreover, EP2^−/−^ mice were more resistant to neurotoxicity induced by MPTP, an effect that is associated with attenuated formation of aggregated *α*-synuclein in the SN and striatum [111].

#### 3.3.3. PGD_2_, PGJ_2_, and Other Prostaglandins

PGJ_2_ and its metabolites might alter the process of protein folding and aggregation, contributing to the development of PD. In human neuroblastoma SK-N-SH cells, PGJ_2_ disrupts the structural integrity of microtubules and actin filaments [112]. *In vitro*, this molecule also hindered the polymerization of highly purified tubulin from bovine brain [113]. Interestingly, in cells treated with PGJ_2_, microtubule/endoplasmatic reticulum collapse coincides with the formation of protein aggregates, such as ubiquitinated proteins and *α*-synuclein [113].

In mouse and human neuroblastoma cells, as well as in rat primary embryonic mesencephalic cultures, PGA_1_, PGD_2_, PGJ_2_, and its metabolite Δ^12^-PGJ_2_ induced accumulation of ubiquitinated proteins and cell death [114]. PGE_2_ only exhibited neurotoxic effects at high concentrations. The ubiquitination induced by Δ^12^-PGJ_2_ might be due to inhibition of ubiquitin C-terminal hydrolase (UCH) L3 and UCH-L1, implicating in an alteration of de-ubiquitinating enzymes, possibly contributing to the accumulation and aggregation of ubiquitinated proteins, what leads to inflammation associated with the neurodegenerative process [114]. Modification of UCH-L1, an enzyme that functions predominantly during monoubiquitin recycling in the ubiquitin-proteosome system, by cyclopentenone prostaglandins, induced unfolding and aggregation of the protein. Therefore, the deleterious effect of COX-2 in PD could be due to the production of cyclopentenone prostaglandins [115].

In addition to that, PGA_1_ has been shown to reduce nuclear factor kappa B translocation to the nucleus, caspase 3 activation, and apoptosis of human dopaminergic SH-SY5Y cells induced by rotenone [116].

### 3.4. Amyotrophic Lateral Sclerosis (ALS)

ALS is a progressive neurodegenerative condition characterized by the selective death of motor neurons [117]. This neuropathological condition can be classified as familial, in which mutations in the enzyme superoxide dismutase-1 (SOD1) can occur, or as sporadic, which encompasses 90% of ALS patients [118]. Neuroinflammation seems to play an important role in the progress of this disorder. In ALS, microglia activation and proliferation is observed in regions where there is neuron loss, like motor cortex, motor nuclei of the brainstem and corticospinal tract. Microglia might be essential for the motor neuron toxicity [119].

#### 3.4.1. PLA_2_ and COX

It has been shown that cPLA_2_ is expressed in astrocytes and motor neurons of the spinal cord of transgenic mice carrying the gene encoding a mutant form of human SOD1 [120, 121]. In agreement with that, cPLA_2_ immunoreactivity was also observed in the spinal cord of human SOD1-mutated familial ALS and in sporadic ALS patients [120, 122].

An increase in COX-2 expression is observed in the spinal cord of SOD1^G93A^ transgenic mice [123, 124] and human cases of ALS [125, 126]. *Postmortem* examination of the ventral horn of the spinal cord of sporadic ALS patients revealed that COX-2 immunoreactivity was increased in motor and interneurons, as well as in glia, in comparison with non-ALS controls [127]. On the other hand, COX-1 expression was detected in microglia, but not in neurons, of ALS and controls tissues, albeit no difference was observed between the two groups of patients [127].

Few attempts have also been made to elucidate the effect induced by COX inhibitors in models of ALS. In organotypic spinal cord cultures, the COX-2 selective inhibitor SC236 significantly reduced the excitotoxic damage of motor neurons induced by threo-hydroxyaspartate, a compound that inhibits astroglial transport of glutamate [128]. Therefore, it is possible that COX-2 might be involved in the excitotoxicity induced by glutamate.

Moreover, *in vivo* studies also suggested that COX might be a potential target for ALS treatment. It has been shown that traditional NSAIDs and COX-2 inhibitors reduced different pathological features developed by SOD1^G93A^ transgenic mice, such as loss of motor neurons and glial activation in the spinal cord, motor impairment and weight loss, as well as these compounds prolonged the survival of the animals [120, 129–131]. Considering these evidences, Minghetti [132] suggested that COX-2 enhancement could be deleterious in ALS not only due to the enhancement of glutamate release by PGE_2_ [133], but also because of the ROS produced by COX peroxidase activity.

On the other hand, Almer et al. [134] have shown a drastically reduced PGE_2_ production in the spinal cord of transgenic SOD1^G93A^/COX-1^−/−^ mice, suggesting a minor role for COX-2 in the production of PGE_2_ in the disease. Moreover, deficiency of COX-1 did not affect motor neuron loss and survival of the animals [134]. These results challenge the concept that COX-2 is the main enzyme involved in ALS.

#### 3.4.2. PGE_2_ and 15d-PGJ_2_

PGE_2_ is elevated in the spinal cord of SOD1^G93A^ mice [130] and in the serum and CSF of ALS patients [127, 135], though the levels of this prostaglandin did not correlate with clinical state of the patients [135].

The role of PGE_2_ was further investigated in *in vitro* models of ALS. In an organotypic spinal cord slice model, motor neuronal death induced by D, L-threo-hydroxyaspartate is reduced by PGE_2_, as well as butaprost and sulprostone, EP2 and EP3 receptor agonists, respectively [136]. Interestingly, in the same study, SC58236, a COX-2 inhibitor, also reduced motor neuron loss.

EP2 receptor expression is increased in astrocytes and microglia of SOD1^G93A^ mice and in astrocytes of human ALS spinal cord. Deficiency of EP2 receptor in SOD1^G93A^ mice increased the survival and grip strength in comparison with SOD1^G93A^/EP2^+/+^ and SOD1^G93A^/EP2^+/−^ mice. The absence of EP2 receptor also reduced the production of different inflammatory mediators in this animal model of ALS [124].

Recently, it has been shown that mPGES-1 is enhanced in the spinal cord of SOD1^G93A^ in comparison with WT mice. Interestingly, AAD-2004, a molecule that inhibits mPGES-1 and free radical formation, reduced microglia activation and motor neuron loss, as well as it improved motor function and increased survival [137].

15d-PGJ_2_ immunoreactivity is increased not only in motor neurons, but also in astrocytes and reactive microglia in the spinal cord of ALS patients [138].

### 3.5. Huntington's Disease (HD)

HD is a progressive neurodegenerative disease that reveals movement disorders and dementia as main features. This pathological condition is an autosomal-dominant pathological condition disease [139, 140]. Although there are evidences that neuroinflammation is present in HD, it is not known whether it contributes to the etiopathogenesis of the disease or whether it is solely an epiphenomenon [141].

It has been shown that in R6/2 mice, an animal model of HD, the number of microglia is reduced in some brain regions in comparison with their WT littermates. Microglia of animals at 14.5 weeks of age were also smaller in size than the same cells in the animals at 7 weeks of age, and they also revealed condensed nucleus and fragmentation of the cytoplasm within processes, suggesting an impaired function of these cells in this pathological condition [142]. On the other hand, activated microglia are present in the neostriatum, cortex, and globus pallidus of HD brains. Importantly, the reactive microglia appeared in association with pyramidal neurons presenting huntingtin-positive intranuclear inclusions [143]. Although a causal link between neuroinflammation and HD onset or progression has not been demonstrated, it is reasonable to assume that microglia might play a role in its development.

#### 3.5.1. COX

Although there are different genetic models of HD, some compounds such as 3-nitropropionic acid (3-NP) and quinolinic acid (QA) are also used to induce striatal neuron toxicity, being therefore considered HD animal models [144–146]. COX-2 immunoreactivity is enhanced in striatal tissues 12 h after treatment of animals with QA. This enhancement was observed predominantly in neurons and microglia [147].

Chronic treatment with different COX inhibitors, such as rofecoxib, celecoxib, nimesulide, and meloxicam improved spontaneous locomotor activity and the motor performance, as well as these medicines reduced biochemical and mitochondrial alteration induced by QA [148–150]. Naproxen and valdecoxib, two COX inhibitors, also reduced 3-NP-induced motor and cognitive impairment [151]. This study suggested that these effects could be due to a reduction in the oxidative stress induced by the drugs.

Although beneficial effects were observed induced by COX inhibitors in drug-induced models of HD, similar effects are not observed in transgenic mice. For example, administration of acetylsalicylate from weaning did not induce any alteration of rotarod performance and ventricle enlargement N171-82Q mice in comparison with untreated animals. Rofecoxib also did not change motor performance and lifespan of R6/2 mice [152]. On the other hand, acetylsalicylate and celecoxib shortened life expectancy of R6/2 and N171-82Q mice, respectively [152, 153].

#### 3.5.2. PGE_2_, PGF_2*α*_, and PGA_1_

Administration of 3-NP enhances PGE_2_ and PGF_2*α*_ in the striatum [154, 155]. These prostaglandins are reduced by licofelone, a competitive inhibitor of COX-1, COX-2, and 5-LOX isoenzymes. In addition, this compound reduced the impairment in locomotor activity and motor performance, as well as it reduced apoptotic markers [155]. Expression of COX-2, as well as PGE_2_ production, is increased in the ipsilateral side compared with the contralateral vehicle-injected side in the striatum and cortex of rats by unilateral intrastriatal injection of QA [156]. Moreover, it has also been shown that QA injection induced EP3-positive striatal neuronal loss, whereas activated microglia expressed EP3 *in vivo* after excitotoxicity injury [157].

A role for PGA_1_ has also been suggested. This prostaglandin attenuated DNA fragmentation and neuronal loss and increased dopamine D1 receptor expression induced by QA in the striatum it also reduced the QA-induced activation of nuclear factor kappa B, but not activator protein-1, in this brain region [158].

## 4. Discussion

There is an intricate relationship between neuroinflammation and neurodegeneration. In general, acute inflammation in the CNS is triggered by a neuronal injury or infection and is short-lived. This acute response is believed to have protective aspects, since it could avoid further injury and induce tissue repair [159]. Although an acute stimulus may trigger, for example, oxidative stress, this short-term event would not interfere with long-term neuronal survival [160]. It is known that moderate microglia activation might induce neuroprotective effects, such as to scavenger neurotoxins, remove cell debris and secrete mediators which are important for neuronal survival [160]. Acute activation of these cells is a normal response to injuries, and it contributes to wound healing [161].

On the other hand, chronic neuroinflammation persists for a long time after the initial insult and normally is self-perpetuating [160]. This condition induces neuronal death, and the molecules released by the dead neurons can further activate microglia, which enhances cell death. This vicious cycle, together with the continuous production of factors that activate microglia, contributes to the chronicity of this process.

 Again, microglia might play an important role in this long-term process. Intense activation and accumulation of these cells at the site of injury can induce neuronal damage, since they release a variety of neurotoxic substances. For example, the A*β* protein, which is involved in AD, can activate microglia and lead them to release neurotoxic factors such as NO, TNF-*α*, and superoxide, leading to the progression of this disorder [162]. An interesting finding is that a chronic inflammation induced by the infusion of LPS (a substance that strongly activates microglia) in the brain of rats resembles different features observed in AD patients [163].

Actually, it is presently not clear why the neuronal or glial cells cannot prevent the chronicity of the inflammatory process. However, it might be due to a plethora of effects. Abnormal synthesis of some proteins by neurons could continuously activate microglia, leading them to the release of neurotoxic factors. Moreover, oxidative stress is another important event that contributes to the neuronal damage observed in chronic neuroinflammation [164]. It is also possible that the senescence of immune system in the CNS could contribute to chronicity of this process. For example, it has been shown that microglia from old transgenic PS1-APP mice release an increased amount of inflammatory mediators and do not phagocytose A*β* properly in comparison to microglia from young mice [165]. Therefore, microglia senescence could play a role in the development of some neurodegenerative conditions [161, 166]. Despite these facts, the adaptive immune system might also play a role, as it has been shown that it is involved in the etiopathogenesis of PD [167].

In this context, one might assume that the production of lipid mediators, such as prostaglandins, might differently modulate neuroinflammation and neurodegeneration. Considering the roles of prostaglandins and depending on the stage of inflammation, as well as different microenvironments generated by a variety of substances, these lipid mediators could determine the survival or death of neurons. 

## 5. Conclusion

Here we summarized the evidences that prostaglandins might play a key role in the etiopathogenesis of neuroinflammatory and neurodegenerative diseases. Prostaglandins have a plethora of actions in CNS cells that differently affect the progress of inflammation and neuronal death or survival. Therefore, inhibition of the production of a specific prostanoid or its action on its receptor would be a better mechanism to control some pathological processes. On the other hand, inhibiting the effects of some prostaglandins could also be deleterious. Thus, further studies are important to make a more complete idea the role of these lipid mediators in neuroinflammation and neurodegeneration. This knowledge might serve to develop pharmacological strategies for the treatment of neurological diseases.

## Abbreviations

15d-PGJ_2_: 15-deoxy-Δ^12,14^-prostaglandin J_2_
3-NP: 3-nitropropionic acid
6-OHDA: 6-hydroxydopamine
AA: Arachidonic acid
AD: Alzheimer's disease
ALS: Amyotrophic lateral sclerosis
A*β*: Amyloid *β*
APP: Amyloid precursor protein
CNS: Central nervous system
COX: Cyclooxygenase
CSF: Cerebrospinal fluid
EAE: Experimental autoimmune encephalomyelitis
HD: Huntington's disease
HPGDS: Hematopoietic prostaglandin D synthase
IL: Interleukin
iNOS: Inducible nitric oxide synthase
KO: Knockout
LPS: Lypopolysaccharide
LTP: Long-term potentiation
MHC: Major histocompatibility complex
MOG: Myelin oligodendrocyte glycoprotein
mOP: Mouse oligodendrocyte precursor
mPGES: Microsomal PGE synthase
MPP^+^: 1-methyl-4-phenylpyridinium
MPTP: 1-methyl-4-phenyl-1,2,3,6-tetrahydropyridine
MS: Multiple sclerosis
NO: Nitric oxide
NSAIDs: Nonsteroidal anti-inflammatory drugs
PD: Parkinson's disease
PG: Prostaglandin
PGIS: PGI synthase
PLA_2_: Phospholipase A_2_
PPARs: Peroxisome proliferator-activated receptors
QA: Quinolinic acid
ROS: Reactive oxygen species
SN: Substantia nigra
SOD1: Superoxide dismutase-1
TNF: Tumor necrosis factor
UCH: Ubiquitin C-terminal hydrolase
WT: Wildtype.
